# Successful Use of a Multidisciplinary Approach to Treat a Perforated Duodenal Malignant Lymphoma in an Elderly Patient

**DOI:** 10.1155/2018/2326459

**Published:** 2018-04-01

**Authors:** Tomoko Takagishi, Yuta Niimi, Goshi Matsuki, Shinta Nagano, Junsuke Hinami, Masaaki Kajiwara, Kiyoshi Kaneko, Yoshihiro Kubota, Osamu Nakai

**Affiliations:** Department of Surgery, Uji Tokushukai Medical Center, Uji, Kyoto 611-0041, Japan

## Abstract

Treatment of duodenal malignant lymphoma is difficult due to life-threatening complications such as intestinal obstruction, perforation, and pancreatitis. Thus, multidisciplinary procedures are required alongside surgical intervention. Contrast abdominal CT images of a 75-year-old female suffering from vomiting revealed thickening of the duodenal wall (from the second to third segment). Gastrojejunostomy and biopsy identified the tumor as diffuse large B-cell lymphoma. A diagnosis of stage II duodenal lymphoma was made. The lymphoma continued to grow, resulting in jaundice and intestinal perforation, which was first treated with two cycles of rituximab and antibiotics. Thereafter, less intensive chemotherapy (two cycles each of R-mini-CHP, CHP, and R-CHOP) was administered, which led to significant improvement upon assessment by PET-CT. Residual lymphoma was treated with consolidation radiotherapy (50 Gy in 25 fractions) over 5 weeks after chemotherapy. The patient attained a complete response and has been disease-free for more than 4 years. Thus, duodenal perforated lymphoma can be treated successfully using a multidisciplinary approach that combines surgery, immunochemotherapy, and radiation therapy.

## 1. Introduction

Primary gastrointestinal lymphoma accounts for 30–40% of all extranodal lymphomas but for only 1–8% of all gastrointestinal malignancies [1]. The majority of malignancies in the duodenum are adenocarcinoma; malignant lymphoma represents only 2% of cases [2]. Malignant gastrointestinal lymphomas increase the risk of stenosis and/or perforation, which is a serious life-threatening complication. Perforation can occur spontaneously due to enlargement of the tumor, or as a consequence of chemotherapy or irradiation [3]. Among the various types of gastrointestinal lymphoma, duodenal lymphoma is rare; however, it is highly morbid [4] and may develop as diffuse large B-cell lymphoma (DLBCL), mucosa-associated lymphoid tissue lymphoma, or Burkitt lymphoma [5, 6]. Duodenal lymphoma tends to be associated with life-threatening complications such as intestinal obstruction, jaundice, pancreatitis, and perforation [6–8]. Perforated gastrointestinal lymphoma increases the risk of peritoneal lymphomatous dissemination [9]. In terms of treatment, emergency surgical interventions are carried out to stall massive bleeding or performed electively to prevent intestinal obstruction or perforation [5]. In addition, recent progress in lymphoma immunochemotherapy means that patients may have a better outcome if treated using a multidisciplinary approach comprising a monoclonal antibody (rituximab, which is specific for CD20-positive B lymphoma cells), less intensive chemotherapy, radiotherapy, and surgical intervention (where needed) [10–13]. Here, we report successful use of a multidisciplinary approach to treat an elderly female patient with duodenal DLBCL complicated by obstruction and perforation.

## 2. Case Report

**Figure 1:**
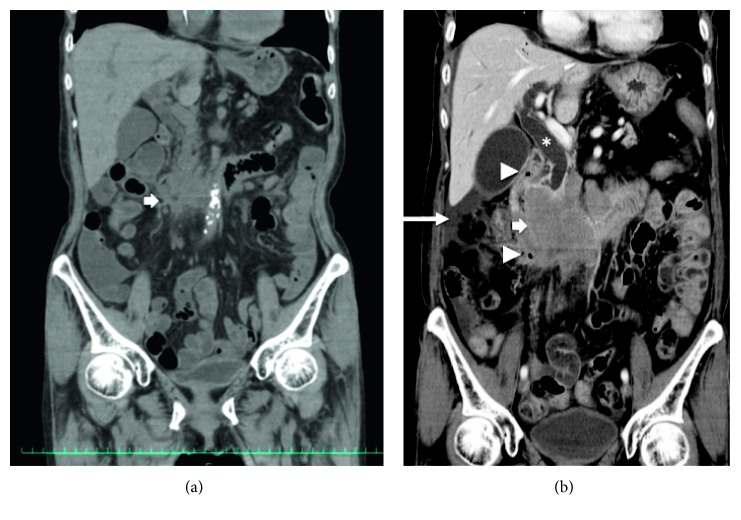
Contrast abdominal CT (coronal) image upon admission. The scan shows (a) prominent thickening of the duodenal wall from the second to third portion (arrow), and (b) perforation 17 days after gastrojejunostomy revealing an enlarged duodenal mass (arrow), free air (arrow heads), ascites (long arrow), and expansion of the common bile duct/intrahepatic bile duct (^∗^) due to tumor-related stenosis.

**Figure 2:**
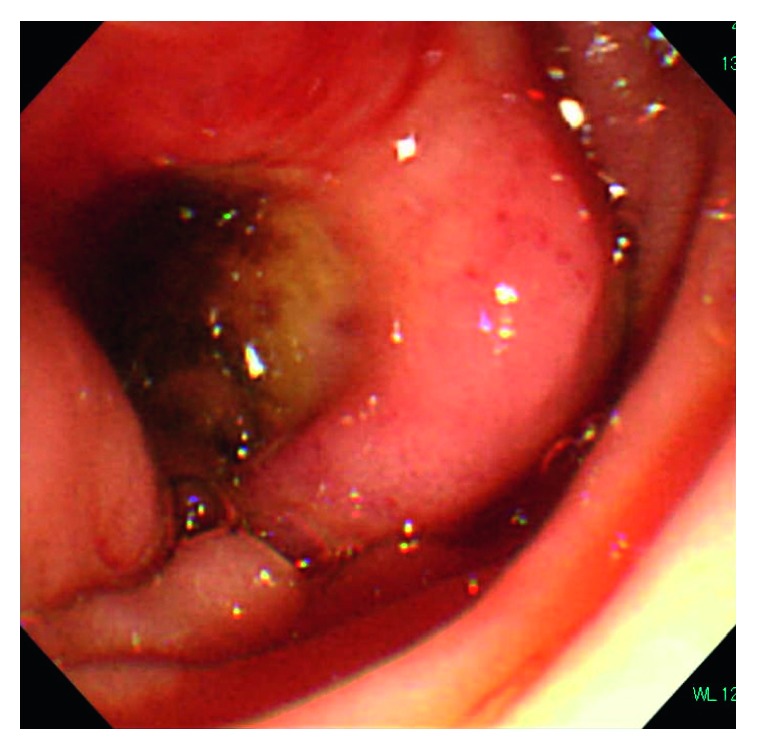
Endoscopic findings. Endoscopy revealed an ulcer and bulging of the thickened duodenal wall (second to third portion), where the perforation occurred.

**Figure 3:**
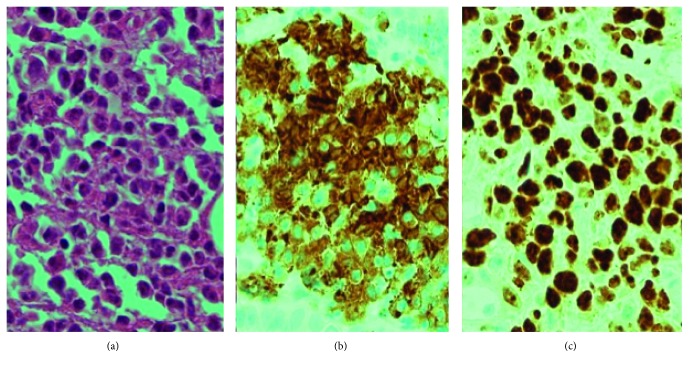
Histopathological findings. A biopsy led to a diagnosis of DLBCL. The tissue was diffusely infiltrated by lymphoblasts with nuclear atypia (a) (H&E stain; original magnification, ×400), which were diffusely stained with CD79a+ (b) and Ki67+ (c) (original magnification, ×400). CD20+ staining not shown.

A 75-year-old female was referred to hospital due to a 5-day history of vomiting and reduced appetite. Over the previous 2 years, she had lost 10 kg. Her past medical history was significant and included hypothyroidism and partial resection of the jejunum due to inflammatory (no malignancy) occlusion. On admission, the patient showed abdominal distention without pain, guarding, or rebound tenderness at the epigastric region. No superficial lymph nodes were palpable. Laboratory findings were as follows: white blood cell (WBC) count, 7,700/*μ*L; hemoglobin (Hb), 10.7 g/dL; platelet count, 543 K/*µ*L; serum lactate dehydrogenase (LDH), 703 U/L; total bilirubin (T-Bil), 1.52 mg/dL; and soluble interleukin-2 receptor (sIL-2R), 757 U/mL. Hepatic function and renal function were normal. Contrast abdominal computed tomography (CT) images showed a mass in the duodenum (Figure 1(a)). Esophagogastroduodenal endoscopy revealed marked thickening and bulging of the duodenal wall from the second to third portion (Figure 2). A diagnosis of duodenal obstruction led to performance of gastrojejunostomy. Histological examination of a biopsy from the duodenal lesion revealed DLBCL (phenotype: CD20+, CD79a+, Ki67+) (Figure 3). Eventually, the patient was diagnosed with stage II duodenal DLBCL.

**Figure 4:**
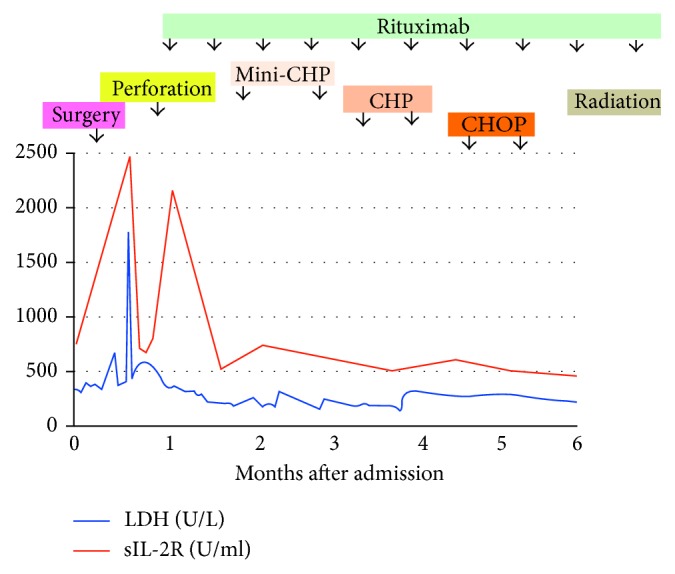
Time course of changes in laboratory data in response to treatment. Serum levels of lactate dehydrogenase (LDH) and sIL-2R in response to duodenal perforation and various therapeutic regimens. Details of treatment regimens are in the text.

Before starting chemotherapy, we planned to have positron emission tomography- (PET-) CT images to confirm the precise stage of lymphoma. Seventeen days after laparotomy during the time waiting for PET-CT, the duodenal mass had enlarged, resulting in obstructive jaundice and perforation of an ulcer in the second to third segment of the duodenum (Figure 1(b)), when the patient complained of severe abdominal pain, resulted in a state of shock with hypotension (76/42 mm Hg) and physical signs suggesting peritonitis. Serum LDH and sIL-2R increased rapidly (Figure 4). The patient was admitted to the intensive care unit and received a vasopressor and antibiotics (ABPC/SBT). In addition, the patient required a nasogastric tube for gastric decompression, along with percutaneous transhepatic biliary drainage to alleviate jaundice, but no need of percutaneous drainage for the periduodenal collection. We chose conservative therapy to manage this case of duodenal lymphoma with perforation. As shown in Figure 4, the patient received two cycles of rituximab, although because she was anti-HBs-Ab-negative and anti-HBc-Ab-positive, we were careful to monitor reactivation of HBV. The mass did not shrink significantly upon treatment with rituximab alone; however, CT revealed healed perforation of the duodenum, and symptoms associated with peritonitis and jaundice were relieved. Thereafter, we started immunochemotherapy by removing vincristine from the CHOP regimen due to concerns about adverse effects on the gastrointestinal system (e.g., paralytic ileus), which might exacerbate perforation. First, the patient received two cycles of R-mini-CHP (rituximab (day 1; 375 mg/m^2^), cyclophosphamide (day 1; 400 mg/m^2^), doxorubicin (day 1; 25 mg/m^2^), and prednisolone (days 1–5; 40 mg/m^2^) in each cycle), with a 40–50% reduction in the dose of doxorubicin, cyclophosphamide, and prednisolone. This was followed by two cycles of R-CHP (rituximab (day 1; 375 mg/m^2^), cyclophosphamide (day 1; 700 mg/m^2^), doxorubicin (day 1; 50 mg/m^2^), and prednisolone (days 1–5; 60 mg/m^2^) in each cycle) with an interval of 21–28 days. Finally, the patient received two cycles of R-CHOP (rituximab (day 1; 375 mg/m^2^), cyclophosphamide (day 1; 700 mg/m^2^), doxorubicin (day 1; 50 mg/m^2^), vincristine (day 1; 1.4 mg/m^2^), and prednisolone (day 1–5; 60 mg/m^2^) on days 1–5 of each cycle) because we thought vincristine could be given at this stage of the treatment. Thereafter, rituximab alone was given as maintenance therapy (total cumulative doses = 14).

In accordance with the Common Terminology Criteria for Adverse Events, grade 3 neutropenia (numbers of event/cycles) was noted during chemotherapy in rituximab alone (*n*=0/14), R-mini CHP (*n*=0/2), R-CHP (*n*=2/2), or R-CHOP (*n*=2/2); however, no peritoneal or bile duct infections were associated with grade 3 neutropenia.

**Figure 5:**
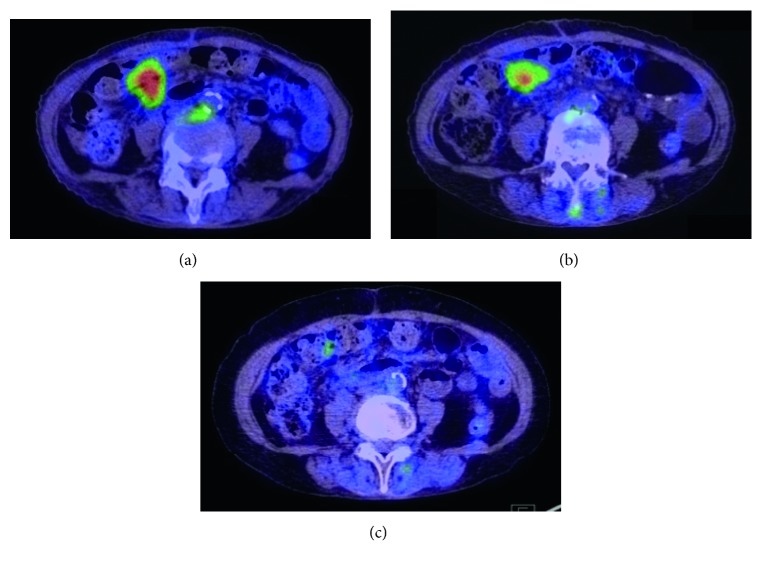
Time course of PET-CT findings regarding the duodenal mass in response to treatment, PET-CT shows (a) high FDG uptake (SUV max = 9.2) at the duodenum prior to immunochemotherapy, (b) reduced uptake (SUV max = 5.1) after radiation, and (c) barely detectable uptake (SUV max <1.0) after finishing treatment.

After completing chemotherapy, the patient's condition improved markedly, although a residual high signal remained in the third segment of the duodenum and in the jejunum upon PET-CT (Figure 5). One month after finishing chemotherapy, the patient received radiotherapy (50 Gy; 25 fractions over 5 weeks) to treat residual lymphoma, after which the hot FDG uptake signal observed on PET-CT images disappeared (Figure 5). No grade 3 neutropenia (numbers of event/fractions) was noted during radiation therapy (*n*=0/25). The patient has remained disease-free for more than 4 years.

## 3. Discussion

Because duodenal lymphoma is rare, there is little information about appropriate therapeutic regimens, especially for elderly patients. The duodenum is one of the most difficult anatomical sites with respect to surgical intervention; thus, radical resection of duodenal lymphoma is rarely performed. Considering the risk of perforation, intensive chemotherapy is not a good idea. Accordingly, a multidisciplinary approach using a combination of rituximab and less intensive chemotherapy followed by radiotherapy was chosen because we felt it would be both effective and safe. Here, we report successful treatment of duodenal lymphoma using this approach.

Sarkhosh et al. discuss surgical intervention for management of duodenal lymphoma [5]. They reviewed 23 cases: eight (35%) treated with surgery alone, eight (35%) with surgery plus chemotherapy, five (22%) with chemotherapy alone, and two (9%) with supportive care. Patients treated with surgery were mostly emergencies, that is, obstruction (58%), perforation (33%), and hemorrhage (8%). Here, we performed gastrojejunostomy prior to immunochemotherapy. The need for radiotherapy in the management of DLBCL in the rituximab era is controversial [13]. If radiotherapy is to be considered for DLBCL, patients with FDG uptake confined to a limited area after primary therapy with R-CHOP are likely to be the best candidates [14]. Indeed, in this case, we used radiotherapy to eradicate residual lymphoma after immunochemotherapy.

Thus, immunochemotherapy plays a key role in the treatment of duodenal lymphoma, which must be both safe and effective. Recently, rituximab has been used either alone or together with other chemotherapeutic agents [10]. Trials of less extensive regimens have also been conducted in frail elderly patients [11, 12]. The risk of paralytic ileus after treatment with vincristine must be borne in mind when treating gastrointestinal lymphomas [15, 16]. In addition, in gastrointestinal lymphomas, half of all perforations occur at the time of initial presentation; the other half occurs after chemotherapy [17–21]. Thus, our regimens were based on a cautious approach to chemotherapy-related paralytic ileus and ensuing perforation, a serious complication occurring in about 9% of gastrointestinal lymphomas along chemotherapy [3]. Here, spontaneous perforation was noted prior to, but not after, chemotherapy.

Gastrointestinal perforation due to lymphoma may extend into sepsis and/or peritoneal dissemination of disease, resulting in a poor outcome and a mortality rate of about 60% [3, 9]. A literature survey revealed reports of four cases of perforated duodenal lymphoma in Japan through 1980–2017 (written in Japanese without English abstracts [22–25]). The clinical characteristics of these four cases plus our own are summarized in Table 1. Of the five cases, three were DLBCL, and the remaining two were T-cell lymphomas. Three patients were alive at the time of reporting, one patient died, and the status of the remaining patient is unknown. In one case, complete resection of the tumor was performed, resulting in complete remission. In another case, minor leakage due to perforation was managed by argon plasma coagulation. Our case was the only one treated using a multidisciplinary approach.

In summary, when treating gastrointestinal lymphoma in the elderly, it is important not to attempt radical surgical resection or start intensive full-scale chemotherapy. We propose the following regimen: first, rituximab alone, followed by less intensive chemotherapy without vincristine. Once the mass size has reduced and the patient's condition has improved, full-scale chemotherapy can be given. If localized lymphoma persists, consolidation radiotherapy is effective at eradicating the residual mass. During treatment, assessment by PET-CT is essential for decision-making. We hope that further studies will confirm the effectiveness of this multidisciplinary approach.

## Figures and Tables

**Table 1:** Cases of perforated duodenal lymphoma reported in Japan.

Number	Reference	Age/sex	Sites of duodenum	Lymphoma type	Time of perforation	Surgery	Chemotherapy	Radiotherapy	Prognosis	Cause(s) of death
1	[22]	65/M	1st	DLBCL	At presentation	Pancreaticoduodenectomy	CHOP	−	Alive 9+ months	−
2	[23]	61/M	4th	T-cell lymphoma	After two cycles of SMILE	Argon plasma coagulation	Etoposide+ CHOP SMILE	−	Unknown	−
3	[24]	82/M	4th	T-cell lymphoma	After 6 cycles of CHOP	Partial resection	CHOP	−	12 months	Abdominal abscess
4	[25]	70/F	2nd	DLBCL	At presentation	Simple closure, peritoneal patch	R-CHOP	+	Alive 304 days	Sepsis
5	Our case	75/F	3rd	DLBCL	Day 21 after surgery, before chemotherapy	Gastrojejunostomy	R R-mini CHP R-CHP R-CHOP	+	Alive 4+ years	−

DLBCL = diffuse large B cell lymphoma; R-CHOP = rituximab (R), cyclophosphamide (C), doxorubicin (H), vincristine (O), prednisolone (P); CHP = cyclophosphamide (C), doxorubicin (H), prednisolone (P); SMILE = steroid (S), methotrexate (M), ifosfamide (I), L-asparaginase (L), etoposide (E).
